# Neural coding of autonomic functions in different states of consciousness

**DOI:** 10.1186/s12984-023-01216-6

**Published:** 2023-07-26

**Authors:** Piergiuseppe Liuzzi, Bahia Hakiki, Maenia Scarpino, Rachele Burali, Antonio Maiorelli, Francesca Draghi, Anna Maria Romoli, Antonello Grippo, Francesca Cecchi, Andrea Mannini

**Affiliations:** 1 Sant’Anna School of Advanced Studies, The BioRobotics Institute, Viale Rinaldo Piaggio 69, 56025 Pontedera, PI Italy; 2 IRCSS Fondazione Don Carlo Gnocchi ONLUS, Via di Scandicci 269, FI 50143 Florence, Italy; 3 Department of Experimental and Clinical Medicine, University of Florence, Largo Brambilla 3, 50143 Florence, FI Italy

**Keywords:** Disorders of consciousness, EEG-ECG mutual information, Brain–heart interaction, Minimally Consciousness State, Quantitative Correlates of Consciousness

## Abstract

Detecting signs of residual neural activity in patients with altered states of consciousness is a crucial issue for the customization of neurorehabilitation treatments and clinical decision-making. With this large observational prospective study, we propose an innovative approach to detect residual signs of consciousness via the assessment of the amount of autonomic information coded within the brain. The latter was estimated by computing the mutual information (MI) between preprocessed EEG and ECG signals, to be then compared across consciousness groups, together with the absolute power and an international qualitative labeling. One-hundred seventy-four patients (73 females, 42%) were included in the study (median age of 65 years [IQR = 20], MCS +: 29, MCS −: 23, UWS: 29). Electroencephalography (EEG) information content was found to be mostly related to the coding of electrocardiography (ECG) activity, i.e., with higher MI (p < 0.05), in Unresponsive Wakefulness Syndrome and Minimally Consciousness State minus (MCS −). EEG-ECG MI, besides clearly discriminating patients in an MCS − and +, significantly differed between lesioned areas (sides) in a subgroup of unilateral hemorrhagic patients. Crucially, such an accessible and non-invasive measure of residual consciousness signs was robust across electrodes and patient groups. Consequently, exiting from a strictly *neuro-centric* consciousness detection approach may be the key to provide complementary insights for the objective assessment of patients' consciousness levels and for the patient-specific planning of rehabilitative interventions.

**Supplementary Information:**

The online version contains supplementary material available at 10.1186/s12984-023-01216-6.

## Introduction

A severe Acquired Brain Injury (sABI), which can be defined as a brain damage related to a pathological event of a non-congenital, perinatal or degenerative nature, such as to determine a coma condition, with Glasgow Coma Scale score—acute phase (GCS) ≤ 8 and lasting more than 24 h, can be provoked by a vascular, traumatic, anoxic, infectious, toxic-metabolic, neoplastic origin, and can cause multiple complex sensory-motor, cognitive and/or behavioral impairments leading to severe disability. Many sABI patients also exhibit a prolonged Disorder of Consciousness (pDoC) which is characterized by either an arousal (i.e., basic reflexes as eyes opening, swallowing, etc.…) or awareness (complex thought processes) alteration due to brain damage of anoxic, traumatic, or vascular aetiology. In this context and within the *continuum* of consciousness [1], coma is characterized by the absence of arousal and awareness, the Unresponsive Wakefulness Syndrome (UWS [2]) by arousal with no awareness and the Minimally Conscious State (MCS [3]) by reproducible but non-consistent awareness (although minimal). Two subcategories, termed MCS + and MCS −, are defined as conditioned to the presence of behavioral evidence of residual higher-order cognitive function [4, 5]. MCS + is defined by the capability of performing command-following and intelligible communication (either verbalization or intentional gestural command), whilst MCS– is assigned when reflexive motor behaviors, object manipulation or localization or visual pursuit is present [4, 6]. Despite the heterogeneity in cognitive, behavioral, and neural profiles that patients exhibit, the need to standardize the assessment of pDoC results in daily consciousness assessments being mostly based on behavioural responses [7], with reference scale the JFK Coma Recovery Scale-Revised (CRS-R) [5]. However, the clinical evaluation of non-reflex behaviors is strongly affected by vigilance fluctuations [8, 9], impairments in the sensory/motor networks [10], associated neurological diseases [11] and diffuse pain [12, 13]. For these reasons, precautions as the repeated administration of the CRS-R [8] and the use of the mirror for the visual pursuit assessment were suggested to reduce the risk of misdiagnosis [14]. However, since the introduction of the concept of covert consciousness [15, 16], neuroimaging assessments are getting a crucial role in the field, providing novel endotypes of non-behavioral consciousness [17, 18] and being suggested as complementary assessments by international guidelines [19, 20]. Nevertheless, the high costs of the assessments and the inherent clinical complexity of pDoC patients reduces the accessibility of neuroimaging techniques.

In this sense and being visceral inputs intrinsically individual and self-specifying (and readily available), recent studies have explored the relationship between consciousness and bio-signals ascending from peripheral tracts controlled by the autonomic system [21, 22]. Thus, moving to the wider problem of consciousness detection and stepping away from a strictly *neuro-centric* approach, authors investigated how autonomic functions could be useful for the diagnosis of consciousness. In this optic, Riganello et al. already provided insights on the prognostic [23] and diagnostic [24] power of Heart Rate Variability (HRV) in patients with a pDoC. In particular, in sedated patients after cardiac arrest, patients with higher HRV complexity were found to be likely to reach favorable outcomes [23]. Moreover, despite a small sample size of sedated patients (14 UWS, 16 MCS patients), the HRV complexity distinguished the two cohorts [24]. On the other hand, no significant differences were found for what concerns heart rate between UWS and MCS patients during resting state [25]. When provided auditory stimuli, the interval between the sound and the subsequent cardiac R-peak was found to be decreased in MCS patients compared to UWS, showing a direct connection between residual brain processing and the consequent modulation of autonomic patterns. Also, Heartbeat-Evoked Responses (HER, corresponding to brain responses to ascending cardiac inputs at each heartbeat) have been shown to correlate with glucose metabolism in the default mode network in the right superior temporal sulcus and in the right ventral occipitotemporal cortex in patients with pDoC [26]. Furthermore, classifiers trained on such HER-related EEG segments improved the accuracy of diagnosis with respect to random EEG segments when diagnosis was performed with FDG-PET assessments (metabolic based) but not when it was assessed via the CRS-R.

It has also been suggested that cortical modulation of peripheral sensory/body functions is influenced by concurrent cognition already in healthy individuals [27, 28]. Pernice et al. [28] reported how mutual information (MI) between peripheral networks and cortical activity decreases with increasing levels of mental stress (rest with respect to arithmetic tests and to sustained cognitive attentive process). Similarly, when analyzing sleep-dependent cortico-cardio interactions, sleep stages have been shown to influence the strength of information transfer between the heart and the brain in healthy individuals [29], increasing with the depth of the sleep. As consciousness level decreases (sleep, anesthesia, various cognitive attentive states), the coupling strength between central and peripheric functions increases, mostly due to an absence of specific cortical processing. Thus, our hypothesis is based on the fact that low cognitive efforts, deep sleep stages, and deep anesthesia levels are characterized by a low-to-none cognitive load as in deepest alterations of consciousness. Consequently, the remaining pDoC brain activity is reasonably related to the functioning of the autonomic system, since its functionality is ensured also during unconsciousness. We speculate that the latter derives from a reduction in cortical processing, hence that the difference in information content between cortical areas and peripheral biological sensors’ signals (i.e., cardiac) increases as the alteration of consciousness becomes more evident. For these reasons, the aim of this work was to first characterize consciousness levels first by means of the EEG absolute power and of the American Clinical Neurophysiology Society (ACNS) terminology for critical care [30] in a large cohort of patients with a sABI. Then, the Mutual Information (MI) [31, 32] between EEG and ECG data was computed electrode-wise and compared across different states of consciousness. Lastly, a secondary analysis was performed on a sub-sample of unilateral hemorrhagic patients, to verify whether the location of the affected lesion influenced EEG-ECG MI values.

## Materials and methods

### Study design and data collection protocol

A prospective observational study was performed enrolling consecutively patients admitted to the IRCSS Fondazione Don Carlo Gnocchi of Florence from 01-01-2020 to 01-03-2022 [33]. Inclusion criteria were diagnosis of an sABI and age > 18. Approval from the local Ethical Committee was obtained (N. 16606_OSS_) and enrollment was done following the Helsinki Declaration. Patients have been included after obtaining written consent signed by a legal guardian. Data concerning demographical (age, sex) and clinical aspects were recorded. Based on at least three consecutive CRS-R evaluations, a clinical diagnosis of consciousness was formulated (UWS, MCS −, MCS +, or E-MCS) both at admission and at discharge following international guidelines [19].

Standard 30-min polygraphy recordings (EEG-ECG) were performed using a digital machine (Gal NT, EBNeuro, Firenze, IT). An EEG prewired head cap, with 19 electrodes (Fp1-Fp2-F7-F8-F3-F4-C3-C4-T3-T4-P3-P4-T5-T6-O1-O2-Fz-Cz-Pz) set according to the 10–20 International Standard System was adopted with previously proposed EEG recording parameters [34] at a sample rate of 128 Hz. ECG recording was performed via electrodes applied to the chest at a sample rate of 128 Hz. Tachogram was extracted using the Python library NeuroKit2 and it was visually inspected for missing beats [35]. Interpolation was used to correct eventual ectopic beats [36].

### EEG preprocessing

Unipolar recordings were re-referenced to the average reference and visually inspected for excessive movement noise. The patient was retained for further analysis if at least 5 consecutive minutes of clean resting-state EEG was present. The initial and endpoint of the longest clean section were manually taken and used to crop the recording. Consequently, recordings were high-pass filtered with a finite-impulse-response zero-phase filter with an Hamming's window at 1 Hz as suggested by the PREP pipeline [37]. Infinite-impulse-response notch (50 Hz) filtering was then applied to further remove power line disturbance. Then, the first five seconds of the recording were discarded to eliminate filtering artifacts. Channels with still excessive or uncorrelated noise were labeled following PREP criteria [37] and interpolated by means of spherical spline interpolation [38, 39] using the MNE library [40].

Lastly, the extended InfoMax Independent Component Analysis (ICA) method was applied to remove artifacts prior to signal reconstruction [41]. Within this step, a pre-whitening Principal Components Analysis (PCA) step was applied, decreasing dimensionality from N_channels_ to N_components_ with N_components_ set to 15. The whitened data, then entered the ICA algorithm. Independent components (ICs) were automatically labeled using the MNE-ICALabel library [42]. In particular, a neural-network classifier trained on crowd-sourced data, based on the Matlab ICLabel implementation [43], was used to select the excluded ICs. Only ICs labeled as brain with a confidence higher than 80% were retained. Channel-level data was then reconstructed from the ICs spaces after including only brain data. Channel-level data was then segmented in epochs with a duration of two seconds with no overlap. Epochs were rejected if their maximal peak-to-peak value exceeded the 0.1–120 μV range or if contained a manual annotation of eyes-opening/involuntary movement of the participant. Then for each electrode, the EEG absolute power was computed in the δ (0.5–3 Hz), θ (3–8 Hz) and α (8–12 Hz) band on epochs of 2 s with no overlap. EEG labeling was also performed with the agreement of two expert neurologists according to the American Clinical Neurophysiology Society (ACNS) terminology [30]. The included descriptors were the presence of an antero-posterior gradient (APG) in the background activity, of a symmetric brain background, the presence of reactivity, and lastly, variability (spontaneous). APG was labeled if, at any point in the epoch, a clear and persistent (> 1 continuous minute) anterior to posterior gradient of voltages and frequencies was present. In particular, lower amplitude and faster frequencies were investigated in anterior derivations while higher amplitude and slower frequencies were investigated in posterior derivations. An EEG recording was labeled as reactive if a change in background EEG activity (amplitude or frequency) was present upon stimulation. Symmetry was defined as present when a consistent asymmetry in amplitude or in frequency was present for at least 50% of the epochs.

### Cortico-cardio interactions

In order to evaluate the amount of shared information content between each EEG channel recording $x(t)$ and ECG signal $e(t)$ a Mutual Information $I(x,e)$ analysis was carried out.

Formally, MI can be stated as follows:

$$I\left(x,e\right)=H\left(x\right)-H\left(x|y\right)$$

with $H\left(x\right)$ the entropy of EEG recordings and $H\left(x|y\right)$ the conditional entropy of $x$ given $y$.

Practically, for non-linear and non-stationary signals, after defining marginal densities of $x\left(t\right)$ and $e\left(t\right)$ as ${\upmu }_{x}\left(x\right)$ and ${\upmu }_{e}\left(e\right)$ and their marginal joint density as $\upmu \left(x,y\right)$, $I(x,e)$ can be practically defined as

$$I\left(x,e\right)=\int \int\upmu \left(x,e\right)log\frac{\upmu \left(x,e\right)}{{\upmu }_{x}\left(x\right){\upmu }_{e}\left(e\right)}\hspace{0.17em}dxdy$$

which results, after binning the continuous time domain in the discrete form, in the following estimate

$$I\left(x,e\right)\approx {I}_{binned}\left(x,e\right)={\sum }_{i,j}p\left(i,j\right)log\frac{p\left(i,j\right)}{{p}_{x}\left(i\right){p}_{e}\left(j\right)}$$

where $${p}_{x}\left(i\right)={\int }_{i}{\upmu }_{x}\left(x\right)\hspace{0.17em}dx$$, $${p}_{e}\left(j\right)={\int }_{j}{\upmu }_{e}\left(e\right)\hspace{0.17em}de$$ and $$p\left(i,j\right)={\int }_{i}{\int }_{j}\upmu \left(x,e\right)\hspace{0.17em}dxde$$.

EEG-ECG MI was computed both after average referencing and Fpz referencing, in order to understand whether the referencing techniques affects its spatial distribution.

### Statistical analysis

Patients with missing data for what concerns consciousness diagnosis, etiology or lesion site were excluded from the analysis. Descriptive statistics were reported in terms of medians and interquartile ranges (IQR) for continuous variables and in terms of counts and percentages for categorical variables. EEG absolute power was tested for normality with the Kolmogorov–Smirnov test. Conditioned to the normality test results, spectral densities entered an ANOVA test (or Kruskal–Wallis test) with dependent variables set to the consciousness states (UWS, MCS −, MCS +, and EMCS). Such procedure for EEG recordings was then repeated for all available channels. Conversely, ACNS-based categorical EEG parameters entered a chi-square analysis. In both cases, conditioned to the significance of the evaluated test, post hoc analyses were carried out. In particular, Dunn-Bonferroni tests were conducted after ANOVA (or Kruskal–Wallis test) and z-tests for multiple comparisons were applied after chi-square analysis. Bonferroni correction was applied to cope with the issue of multiple comparisons.

Mutual information computed for each ECG-EEG channel pair was compared within consciousness states via ANOVA or Kruskal–Wallis tests (conditioned to normality results) and consequently entered Dunn-Bonferroni tests conditioned to previous significance. Also, spatial heterogeneity was tested for the whole cohort and the individual consciousness groups with a Kruskal–Wallis test where the electrode location was set as the grouping variable. Then, restricting the analysis to hemorrhagic patients only (for sample size reasons), a comparison was performed between lesioned and non-lesioned areas and between different consciousness states. To do so, a 2-way ANOVA was performed to allow for both main and interaction effects. The data that support the findings of this study are available for research purposes (Additional files 1, 2).

## Results

### Cohort

From an initial cohort of 189 sABI patients, 15 were excluded due to missing clinical data. Thus, one hundred seventy-four patients (73 females, 42%) were included in the study with a median age of 65 years [IQR = 20] and median CRS-R value of 18 points [IQR = 14] (Table 1). Out of 174, 81 (46.6%) patients were diagnosed with a pDoC (MCS +: 29, MCS −: 23, UWS: 29). The patient’s etiologies resulted distributed as follows: 39 patients with a traumatic brain injury, 12 anoxic, 32 ischemic, 79 hemorrhagic patients and 10 patients with other etiology (cancer, encephalitic, infectious or mixed). In particular, in the overall cohort, 96 patients suffered from a mono-lateral lesion (44 right, 52 left) and respectively 33, 30, and 15 from a bilateral, sub-tentorial, or diffuse one (Table 1).

**Table 1:** Cohort clinical and demographic description

	Descriptive statistics
Age, years	65 [20]
Sex, F	73 (42)
Etiology	
Traumatic	39 (22.4)
Anoxic	12 (6.9)
Ischemic	32 (18.4)
Hemorrhagic	79 (45.4)
Other	10 (5.2)
Lesion site	
Right hemisphere	44 (25.3)
Left hemisphere	52 (29.9)
Bilateral	33 (19)
Sub-tentorial	30 (17.2)
Diffuse axonal injury	15 (8.6)
Clinical state	
EMCS	93 (53.4)
MCS +	29 (16.7)
MCS −	23 (13.2)
UWS/VS	29 (16.7)
CRS-R, points	18 [14]

Median [interquartile range] is reported for numerical variables using brackets while counts (percentages) for categorical variables using parenthesis

*EMCS* Emergence from Minimally Consciousness State, *MCS* Minimally Consciousness State, *UWS* Unresponsive Vegetative State, *CRS-R* Coma Recovery Scale—Revised

### Qualitative and quantitative EEG

Using the American Clinical Neurophysiology Society Critical Care classification, the presence of an anteroposterior reorganization of brain activity (p = 0.013, χ^2^ = 16.156) and the presence of cortical reactivity (p = 0.001, χ^2^ = 28.182) resulted significantly correlated with a better consciousness state. In particular, Bonferroni corrected z-tests for multiple comparisons showed how a significantly lower percentage of EMCS patients (6.5%) did not present cortical reactivity with respect to the MCS − (30.4%) and the UWS (34.4%) groups. Similarly, the presence of sustained cortical reactivity was found to be much rarer in the UWS cohort (20.7%) with respect to the patients who exited a pDoC (52.7%) opposed to its complete absence (UWS: 27.5%; EMCS: 4.3%). On the other hand, the presence of brain symmetry was found to be unrelated to consciousness states in such groups with heterogeneous aetiology (Table 2).

**Table 2:** ACNS descriptors

ACNS parameters	N (%)	p-value	χ^2^	EMCS (N = 93)	MCS + (N = 29)	MCS −(N = 23)	UWS (N = 29)
APG		0.013	16.156				
Absent	29 (16.7)			8^a^	4^a, b^	7^b^	10^b^
Present	131 (75.3)			78^a^	21^a^	14^a^	18^a^
N/A	14 (8)			7^a^	4^a^	2^a^	1^a^
Reactivity		0.001	28.182				
Present	70 (40.2)			49^a^	8^a, b^	7^a, b^	6^b^
Not constant	74 (42.5)			32^a^	16^a^	12^a^	14^a^
Not clear	14 (8.0)			8^a^	4^a^	1^a^	1^a^
Absent	16 (9.2)			4^a^	1^a, b^	3^a, b^	8^b^
Symmetry							
Present	96 (55.2)	0.169	5.049	–	–	–	–

Descriptive statistics (count, percentages). Chi-square analysis of ACNS parameters with respect to consciousness state and related z-test for multiple comparisons. Each subscript letter (^a, b^) denotes a subset of consciousness state categories whose column proportions do not differ significantly from each other at α = 0.05 level with the Bonferroni correction

*ACNS* American Clinical Neurophysiology Society, *APG* Antero-Posterior Gradient, *EMCS* Emergence from Minimally Consciousness State, *MCS* Minimally Consciousness State, *UWS* Unresponsive Vegetative State

**Fig. 1:**
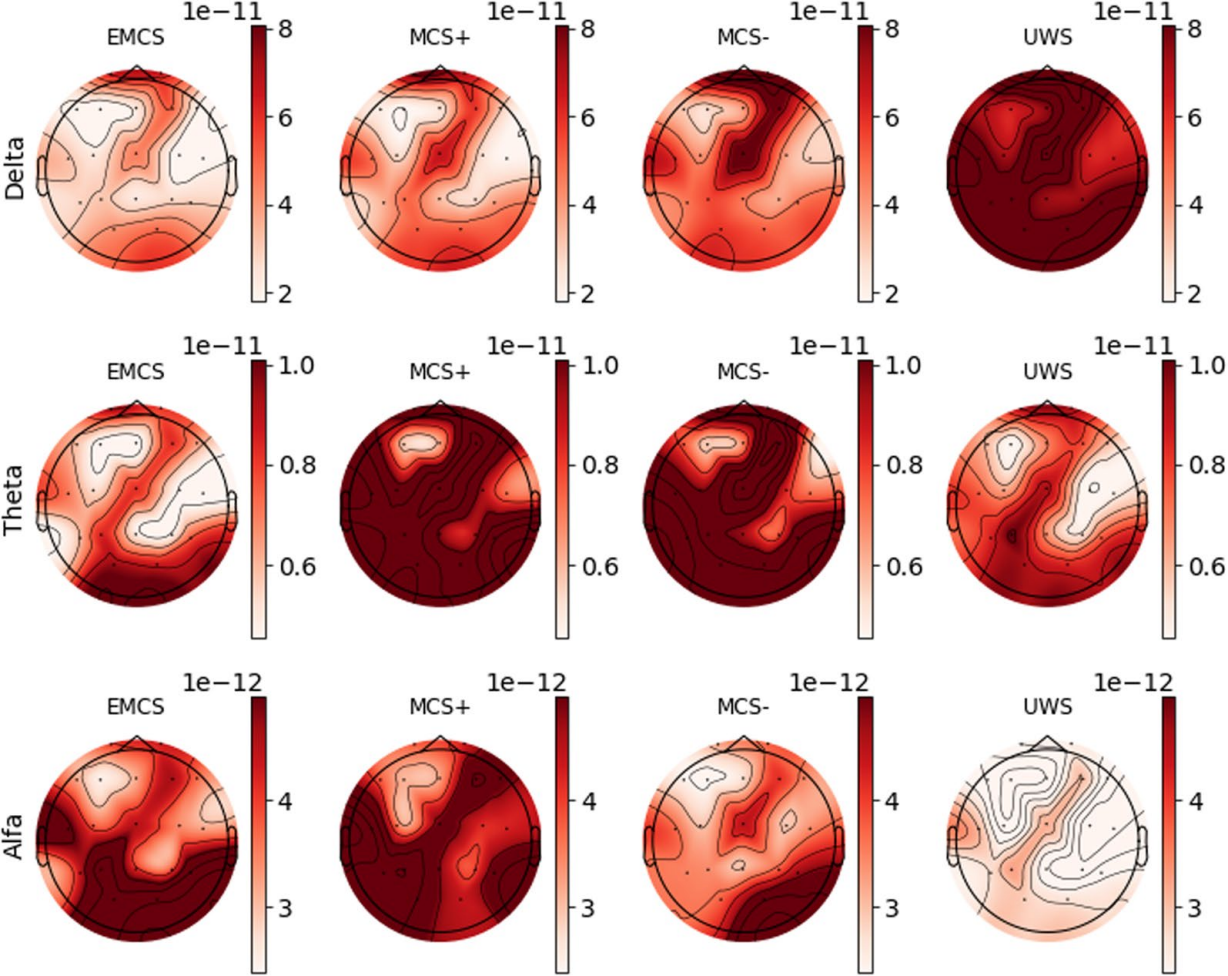
EEG Power Spectral Density across consciousness states. Absolute power is reported on the scalp for each consciousness state (columns) and each frequency band (rows). The topographic map is obtained by performing a spatial 2D interpolation of the PSD values at each electrode (median across patients of that group)

Power spectral densities were computed in the delta, theta, and alpha bands and grouped across consciousness states (Fig. 1). $PS{D}_{\updelta }$ was found to be increasing while going towards worse consciousness levels. Conversely, $PS{D}_{\mathrm{\alpha }}$ was found to be higher in EMCS and MCS + cohorts. Kruskal–Wallis analysis applied to single-electrode PSD showed how, independently from the area the signal is recorded from, $PS{D}_{\uptheta }$ is significantly different across consciousness states (Additional file 3). In particular, Dunn-Bonferroni post-hoc tests showed how patients in an EMCS or MCS + retained significantly less $PS{D}_{\updelta }$ than patients with a UWS for all electrodes, except for Fp1 and Fp2. Also, significantly higher $PS{D}_{\updelta }$ (p < 0.05) was detected in the UWS group compared to MCS − group on the frontal line (F7-F3-Fz-F4-F8). For what concerns $PS{D}_{\uptheta }$, differences were found between the EMCS group and the MCS +/MCS − cohorts. In both cases, after the Bonferroni corrections, only some electrodes retained their significance (Cz, T3, T4 and T5 for the EMCS/MCS + pair and F3-F4-F8-C4-P3-Pz-T4 for the EMCS/MCS − pair, Additional file 3). Substantial differences were found between EMCS and both MCS −/UWS groups for what concerns $PS{D}_{\mathrm{\alpha }}$ with almost all electrodes showing a significant difference between the pairs also after the Bonferroni correction (Additional file 3).

### MI across consciousness states

**Fig. 2:**
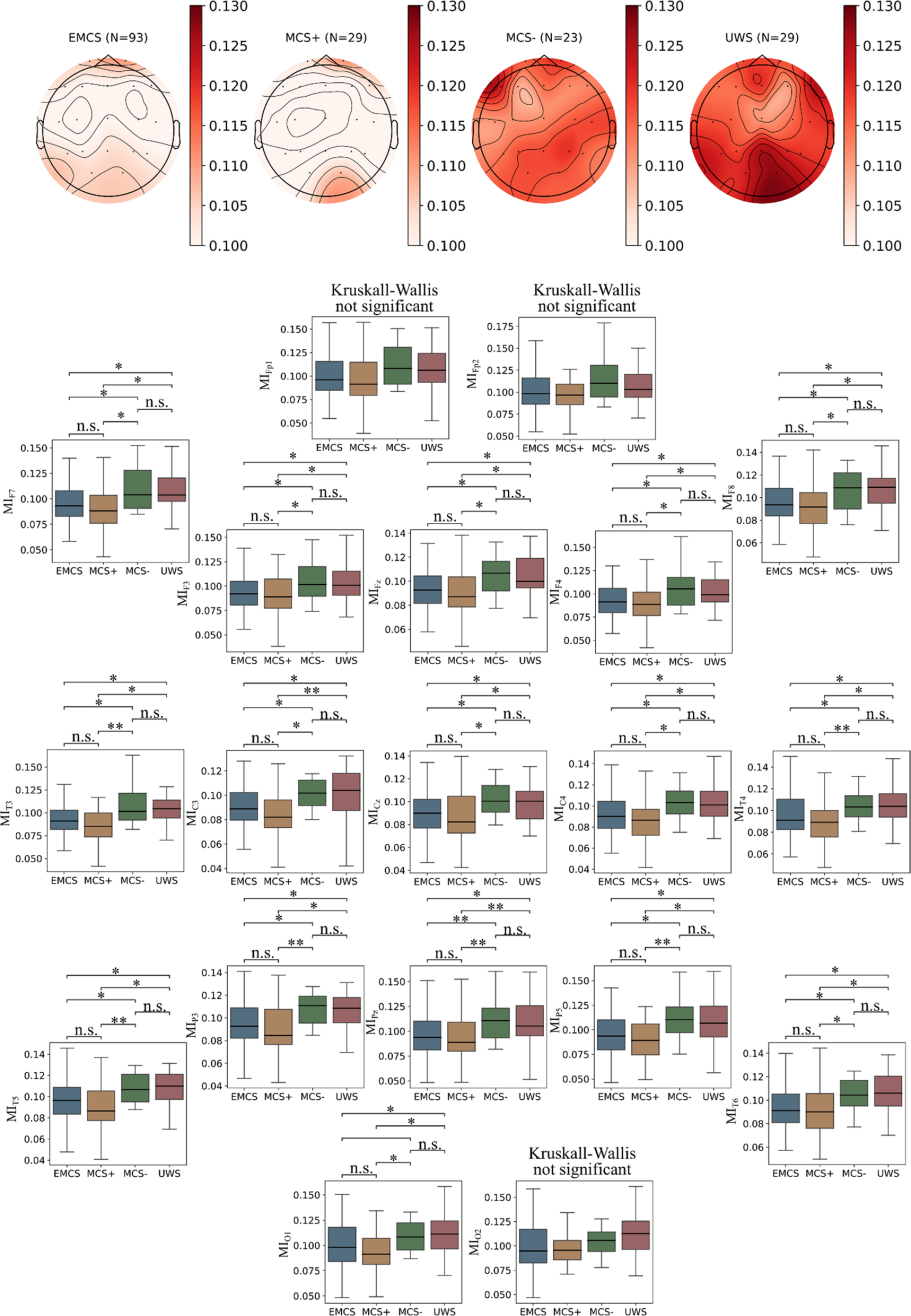
EEG-ECG Mutual Information across consciousness states. Spatial interpolation of median (across patients) MI values for each consciousness group (upper subplots). Box-plots with electrode-wise groups comparisons of MI across consciousness states (lower subplots) with related pair-wise Dunn-Bonferroni significances (conditioned to Kruskal–Wallis significance). Subplots are distributed in space following approximate position within the 10–20 system

Results of the computation of MI across different consciousness states are reported in a topographic map showing for each electrode the median value of the specific electrode across patients within that group (Fig. 2) and the data used is provided within Additional file 4. This was repeated by referencing the EEG signal to Fpz and the results of this section computed on average reference were reported in Additional file 4.

Visually, upper levels of consciousness (EMCS and MCS +) are clearly lower in ECG-EEG MI than lower levels of consciousness (MCS − and UWS). Also, electrode-wise, Kruskal–Wallis tests resulted in the MI of electrodes on the frontal, central and parietal lines being significantly different across groups.

In particular, a robust and consistent difference is found between EMCS and both MCS −/UWS groups and between MCS + and both MCS −/UWS groups (p < 0.05, after Bonferroni, Fig. 2B). Only fronto-polar and occipital electrodes were not found to be significantly different at the group level. Heterogeneity of the MI spatial distribution was tested for all groups together (Kruskal–Wallis with grouping variable set to the electrode location) and within each consciousness group. In the first case, a significant group difference was detected (p < 0.001) but the only pair-wise post-hoc significances were found between the Fp2 electrode and Fz (p = 0.024), Cz (p = 0.001) and C3 (p = 0.028). Similarly, for the EMCS group (p = 0.049 at the group level), no pair-wise comparisons survived the Bonferroni correction, preventing from rejecting the hypothesis on MI distribution homogeneity. For what concerns the MCS +, MCS − and UWS groups no significant differences were detected at the group level (respectively p = 0.962, p = 0.745, p = 0.960).

### MI across hemorrhagic sites

**Fig. 3:**
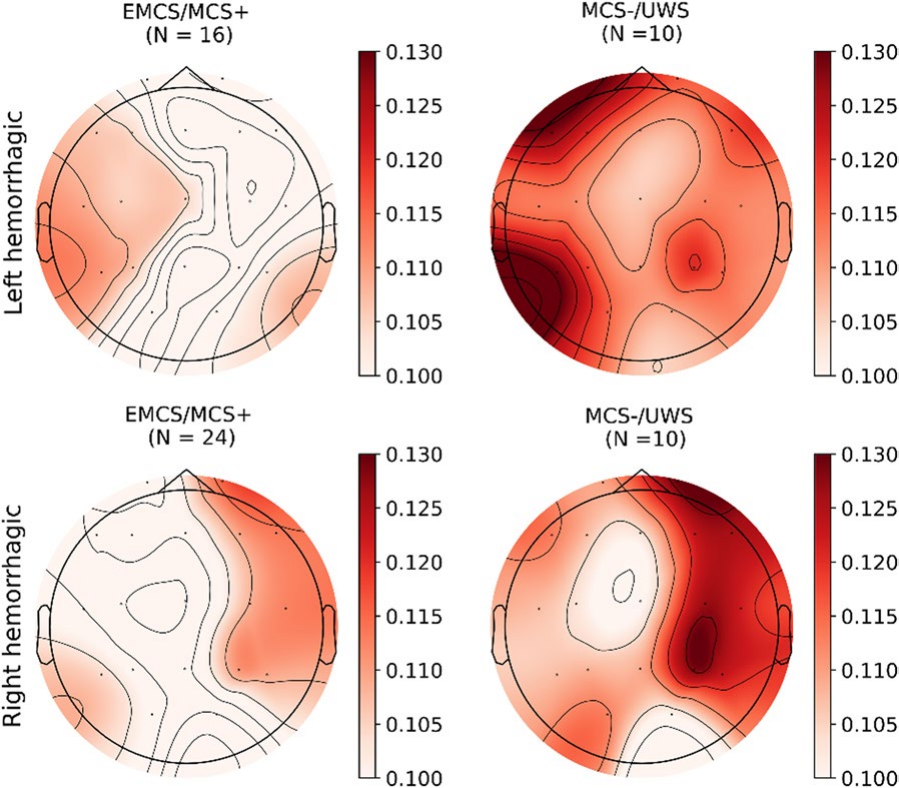
EEG-ECG Mutual Information across hemorrhagic sites. Spatial interpolation of median (across only hemorrhagic patients) MI values for higher and lower consciousness levels (left versus right) and for each lesion side (top versus bottom)

Of the 79 hemorrhagic patients, 60 suffered from a unilateral lesion (26 left and 34 right), 9 from a bilateral lesion, and 11 from a sub-tentorial hemorrhage. Conditioned to the results of the previous analysis and to the inherent similarity between MCS + and EMCS patients, such cohorts were grouped together as well as patients in an MCS − and UWS (Fig. 3).

For each electrode, a 2-way ANOVA analysis was conducted to evaluate the combined effect of the lesion side and consciousness state on the MI. In particular, for almost all electrodes simple main effects analysis showed that the lesion side did have a statistically significant effect on MI (p < 0.05, Table 3). Only the electrodes T5 and P3 did not reach statistical significance, still though retaining a weak effect on MI (respectively F = 3.935; p = 0.054 and F = 3.362; p = 0.081). In particular, the electrodes located within the side with the lesion resulted in a constantly higher MI with the ECG signal. Similarly, for all electrodes except (F8, T3, T4) consciousness level resulted in a statistically significant main effect on the MI (p < 0.05). The analysis also revealed that there was not a statistically significant interaction between the effects of lesion side and consciousness levels.

**Table 3:** Influence of lesion side and consciousness on MI

2-way ANOVA	Source	F	p-value
Fp1	Side	4.212	**0.045**
	Consciousness	9.168	**0.004**
	Side*Consciousness	2.267	0.138
Fp2	Side	14.429	**< 0.001**
	Consciousness	2.205	0.143
	Side*Consciousness	0.152	0.698
F7	Side	5.874	**0.021**
	Consciousness	4.152	**0.046**
	Side*Consciousness	0.251	0.618
F3	Side	6.201	**0.013**
	Consciousness	5.312	**0.025**
	Side*Consciousness	1.005	0.320
F4	Side	18.151	**< 0.001**
	Consciousness	4.824	**0.032**
	Side*Consciousness	0.065	0.799
F8	Side	12.177	**0.001**
	Consciousness	1.958	0.167
	Side*Consciousness	0.118	0.733
T3	Side	4.144	**0.047**
	Consciousness	3.189	0.080
	Side*Consciousness	1.011	0.319
C3	Side	4.162	**0.046**
	Consciousness	4.570	**0.037**
	Side*Consciousness	1.970	0.166
C4	Side	20.306	** < 0.001**
	Consciousness	6.751	**0.012**
	Side*Consciousness	0.166	0.685
T4	Side	14.557	**< 0.001**
	Consciousness	2.580	0.114
	Side*Consciousness	0.089	0.766
T5	Side	3.935	0.054
	Consciousness	5.090	**0.028**
	Side*Consciousness	0.710	0.403
P3	Side	3.362	0.081
	Consciousness	4.412	**0.040**
	Side*Consciousness	1.614	0.209
P4	Side	13.191	**0.001**
	Consciousness	6.772	**0.012**
	Side*Consciousness	0.484	0.490
T6	Side	13.603	**0.001**
	Consciousness	4.601	**0.036**
	Side*Consciousness	0.527	0.471

Results on secondary analysis on hemorrhagic patients only. UWS, MCS − and MCS +, e-MCS patients are respectively grouped together. For each electrode a 2-way ANOVA was performed with the main effects represented by side (left versus right) and consciousness (EMCS/MCS + versus MCS − /UWS). Variable in bold were considered significant

## Discussion

Detecting residual signs of consciousness is one of the most challenging issues in the neurological sciences of the past two decades rising a number of clinical, economic and ethical questions [44]. In this context, a behavioral definition of the consciousness, as recommended by the current guidelines, allowed the standardization of its clinical assessment and the reduction of misdiagnosis [19, 45, 46]. Nevertheless, a decade of research has provided overwhelming evidence that consciousness can occur in pDoC patients also in the complete absence of intentional behavior [7, 46] leading to a revision of the current semiotic classification of consciousness [17, 18].

Thus, for what concerns the first aim of this work, we evaluated spectral power in fixed frequency bands together with EEG descriptors derived from the American Clinical Neurophysiology Society’s (ACNS) Standardized Critical Care EEG Terminology [30]. Precisely, delta and alpha power were found to be indicators of respectively lower and higher levels of consciousness, coherent with literature [34, 47, 48]. UWS patients differed from all upper consciousness levels for what concerns delta power. Such results are in line with Fingelkurts et al. [49] who found, via probability-classification analysis, that the likelihood of delta oscillations occurrence was higher in the UWS patients compared to MCS ones, confirming that a loss of consciousness is associated with slower EEG oscillations. Conversely, they also found that fast alpha oscillations had a higher probability of occurring in MCS patients with respect to UWS patients. Interestingly, based on alpha waves, EMCS patients differed from MCS − and UWS patients but not with the MCS + cohort suggesting that, in contrast to behavioral classification of consciousness, MCS − have an electrical pattern more similar to UWS than MCS +. However, in our analysis, neither power spectra in band-specific analysis nor ACNS parameters were capable to precisely distinguish MCS − and MCS + groups.

As underlined in recent systematic reviews [51–53], some studies based on brain imaging techniques investigated the instrumental stratification of consciousness states exploring the differentiation between MCS − and MCS +. [54–58] Such differential assessment is crucial for medical decision-making, communication with relatives, and rehabilitation pathway planning, given the better prognosis in recovering full consciousness for MCS + patients [6, 57]. Nevertheless, despite such encouraging results, these sophisticated techniques are often limited in feasibility and translatability in every-day clinical practice. In contrast, EEG has the advantage of being non-invasive, inexpensive, and easily repeatable for multiple and long-time assessments [34, 59, 60]. To our knowledge, only two studies have derived an EEG marker to differentiate MCS + from MCS −. Rizkallah et al. [58] in patients with pDoC found that the participation coefficient in theta band was higher in the MCS + group with respect to the MCS − one, although it did not survive the false discovery rate correction. Also, Chatelle et al. showed how the Synek’s criteria for EEG background and reactivity can discriminate 5 MCS + patients from 3 MCS − ones [55].

In our work, we speculated that, since the lowest levels of consciousness are characterized by a low-to-none higher order cognitive activity, the majority of their brain activity is entitled to the maintain the correct functioning of the autonomic system. Such hypothesis is supported by previous findings where different states of awareness and arousal are tested via computation of cortico-cardio interactions. Shiogai et al. investigated how cortico-cardio-respiratory interactions vary during different anesthesia levels in rats by means of computations of Granger causality between peripheric and central networks [61]. The authors highlighted how a significant decrease of network causality is found after the transition from deep to light pentobarbital anesthesia (Fig. 4 of Shiogai et al.). Also, MI between peripheric and central sources was evaluated at different levels of mental stress in healthy individuals. In particular, the MI between brain and body (RR intervals, respiratory intervals and cardiovascular activity) did not show a clear spatial heterogeneity, similar to our findings in patients with a pDoC. Coherently with our findings, it has been shown how EEG-ECG MI decreases from rest to a mental engagement (arithmetic test) to a game (7-min sustained attention) [28].

Additional support to our hypothesis comes from the sleep studies. In particular, Yang et al. [29] explored the sleep-dependent directional interactions of the CNS with the cardio-respiratory network in three main sleep stages, reporting an information transfer decrease of heart rate-to-brain and respiration rate-to-brain information transfer while going from deep to light to REM sleep. This shows how, as the depth of the sleep increases, the information content within the brain mostly relates to coding of autonomic functions.

In short, our results confirm what has already been observed in comparable clinical conditions (e.g., sleep, anesthesia [62]), confirming our hypothesis that in altered states of consciousness neural coding contains mostly information on autonomic signals. Also, differently from what the current behavioural taxonomy suggests, our results provide evidence of a significant difference between MCS + and MCS −, suggesting a possible association (for what concerns central-autonomic modulation) of the latter in a *lower* consciousness level together with UWS. Combining UWS and MCS − together, allowed us to perform a secondary analysis and confirm that EEG-ECG MI is also related to a structural deficiency of the brain (due to the lesion), and where most likely it could be optimally computed. However, the absence of neuroimaging scans of the patients did not allow to precisely map the EEG-ECG MI on the lesion topography, nor to compute any spatial correlation. As fundamental limitation of the proposed approach and one of its main further outlooks, one must consider that MCS − and UWS do no share neither behavioral nor covert correlates of consciousness, thus a greater sample size would allow to perform etiology-specific analysis of MI conditioned to various lesion sites and sizes.

Among the limitations of the work, also the mono-centric nature of the study must be acknowledged which inherently calls for external validations. However, the large sample size used and the evidence obtained from the secondary analysis allows us to moderately infer results to the general population. Also, whether high-density EEG set-ups may increase the diagnostic performances of EEG-ECG MI in the differential diagnosis of MCS ± has to be tackled in future studies, together with the combined use of resting-state and stimulus-based protocols. However, the fact that already 19-channels set-ups accurately discriminate the two cohorts paves the way for minimal, easily translatable configurations, also usable bed-side (fundamental in patients with a pDoC).

In conclusion, our work reveals a shared link between the presence of higher-order cognitive capabilities/behavioral responses and the relative amount of autonomic information coded in the brain. Such inexpensive, non-invasive proxy of residual cognitive processing opens a new window on the differential diagnosis of levels of consciousness, without using less accessible techniques as fMRI/PET or invasive stimulations such as TMS/tDCS. These results show a profitable way to complement consciousness assessment and its related alterations which must leave a rigidly *neuro-centric* approach, paving the way for a multimodal instrumental diagnosis.

## Supplementary Information

**Additional file 1.** The data that support the main analysis of this study, including clinical, EEG power spectrum and EEG-ECG Mutual Information.

**Additional file 2.** The file includes EEG-ECG Mutual Information data supporting the secondary analysis on hemorrhagic patients.

**Additional file 3.** Results of statistics comparisons of PSD estimates in δ, θ, and α bands among consciousness states with post-hoc analysis (FDR corrected).

**Additional file 4.** Reproduction of Figures 2 and 3 with re-referencing to Fpz.

## Data Availability

The data that support the findings of this study are openly available in Additional file 1 for the main analysis and in Additional file 2 for the secondary analysis on hemorrhagic patients. EEG recordings can be provided to researchers for replication purpose upon request to the corresponding author.

## Abbreviations

pDoC: Prolonged Disorders of Consciousness
UWS: Unresponsive Wakefulness Syndrome
MCS: Minimally Conscious State
CRS-R: Coma Recovery Scale-Revised
FDG-PED: FluoroDeoxyGlucose-Positron Emission Tomography
MI: Mutual information
HRV: Heart rate variability
HER: Heartbeat-evoked responses
sABI: Severe acquired brain injury
ICA: Independent component analysis
PCA: Principal component analysis
ACNS: American Clinical Neurophysiology Society
APG: Antero-posterior gradient
PSD: Power spectral density
MMN: MisMatch Negativity
