# Supplementary material for: Neural coding of autonomic functions in different states of consciousness
Source: J Neuroeng Rehabil. 2023 Jul 26;20:96. doi: 10.1186/s12984-023-01216-6 (PMC10369699; doi:10.1186/s12984-023-01216-6)
Supplement: Supplementary file 3 — Additional file 3. Results of statistics comparisons of PSD estimates in δ, θ, and α bands among consciousness states with post-hoc analysis (FDR corrected). [file 12984_2023_1216_MOESM3_ESM.pdf]

Supplementary material B

PSD_{δ}	p-value	χ	EMCS MCS+	EMCS MCS-	EMCS UWS	MCS+ MCS-	MCS+ UWS	MCS- UWS
Fp1	**0.002**	14.958	1.000	0.222	**0.002**	0.788	0.053	1.000
Fp2	**0.010**	11.392	1.000	0.192	**0.020**	0.760	0.216	1.000
F7	**<0.001**	43.935	1.000	0.272	**<0.001**	1.000	**<0.001**	**0.005**
F3	**<0.001**	61.145	1.000	0.121	**<0.001**	0.430	**<0.001**	**0.001**
Fz	**<0.001**	46.272	1.000	0.221	**<0.001**	0.850	**<0.001**	**0.006**
F4	**<0.001**	21.803	1.000	0.101	**<0.001**	0.400	**0.038**	**0.010**
F8	**<0.001**	36.639	1.000	0.054	**<0.001**	0.913	**0.001**	**0.038**
C3	**<0.001**	34.950	1.000	0.093	**<0.001**	0.486	**<0.001**	0.134
Cz	**<0.001**	24.731	1.000	0.070	**<0.001**	0.109	**0.004**	1.000
C4	**<0.001**	49.301	1.000	0.152	**<0.001**	0.953	**<0.001**	0.004
T4	**<0.001**	54.809	1.000	0.067	**<0.001**	0.265	**<0.001**	0.059
T5	**<0.001**	39.100	1.000	0.057	**<0.001**	0.288	**<0.001**	0.154
P3	**<0.001**	26.253	1.000	0.886	**<0.001**	1.000	**0.003**	0.234
Pz	**<0.001**	38.879	1.000	0.263	**<0.001**	1.000	**<0.001**	0.123
P4	**<0.001**	41.950	1.000	0.773	**<0.001**	0.413	**<0.001**	0.057
T3	**<0.001**	26.295	1.000	0.068	**<0.001**	0.904	**0.008**	0.662
O1	**<0.001**	26.639	1.000	0.741	**<0.001**	1.000	**0.001**	0.058
O2	**<0.001**	20.661	1.000	1.000	**<0.001**	1.000	**0.004**	0.017
T6	**<0.001**	33.613	1.000	0.193	**<0.001**	1.000	**<0.001**	0.067

PSD_{θ}	p-value	χ	EMCS MCS+	EMCS MCS-	EMCS UWS	MCS+ MCS-	MCS+ UWS	MCS- UWS
Fp1	0.098	6.303	--	--	--	--	--	--
Fp2	0.076	6.877	--	--	--	--	--	--
F7	0.054	7.651	--	--	--	--	--	--
F3	**0.042**	8.218	**0.053**	**0.042**	1.000	1.000	0.965	0.491
Fz	0.062	7.351	--	--	--	--	--	--
F4	**0.004**	13.412	**0.059**	**0.021**	1.000	1.000	0.142	0.240
F8	**0.002**	14.421	**0.063**	**0.007**	1.000	1.000	0.073	0.300
C3	**0.028**	9.063	0.180	0.116	1.000	1.000	0.382	0.458
Cz	**0.014**	10.588	**0.040**	0.145	1.000	0.294	0.783	1.000
C4	**0.001**	15.466	0.070	**0.018**	1.000	1.000	0.023	0.062
T4	**0.010**	11.337	**0.044**	0.100	1.000	1.000	0.409	0.201
T5	**0.006**	12.443	**0.025**	**0.051**	1.000	1.000	1.000	0.754
P3	**0.029**	9.051	0.788	**0.027**	1.000	1.000	0.327	1.000
Pz	**0.006**	12.453	0.161	**0.011**	1.000	1.000	0.209	0.868
P4	0.121	0.582	--	--	--	--	--	--
T3	**0.002**	14.477	**0.040**	**0.014**	1.000	1.000	0.137	0.222
O1	0.235	4.257	--	--	--	--	--	--
O2	0.250	4.107	--	--	--	--	--	--
T6	0.095	6.373	--	--	--	--	--	--

PSD_{α}	p-value	χ	EMCS MCS+	EMCS MCS-	EMCS UWS	MCS+ MCS-	MCS+ UWS	MCS- UWS
Fp1	**0.015**	10.429	1.000	0.084	**0.021**	0.985	0.712	1.000
Fp2	**0.035**	8.628	0.978	0.148	**0.027**	1.000	1.000	1.000
F7	**0.021**	9.695	1.000	0.060	**0.044**	0.722	0.959	1.000
F3	0.135	5.563	--	--	--	--	--	--
Fz	**0.026**	9.289	1.000	**0.072**	**0.049**	0.780	0.992	1.000
F4	**0.010**	11.252	1.000	**0.014**	**0.039**	0.473	1.000	1.000
F8	**0.002**	14.582	0.702	**0.001**	**0.021**	0.346	1.000	0.616
C3	**0.012**	10.925	0.743	**0.030**	**0.014**	1.000	1.000	1.000
Cz	0.106	6.127	--	--	--	--	--	--
C4	**0.002**	14.464	0.918	**0.002**	**0.025**	0.268	1.000	0.600
T4	**0.008**	11.885	0.299	**0.004**	**0.045**	1.000	1.000	0.621
T5	**0.030**	8.930	1.000	**0.020**	0.175	0.952	1.000	0.881
P3	**<0.001**	18.589	1.000	**0.011**	**0.004**	0.055	0.039	1.000
Pz	**0.001**	15.939	1.000	**0.006**	**0.014**	0.064	0.199	1.000
P4	**0.002**	14.899	0.510	**0.004**	**0.003**	0.847	1.000	1.000
T3	**0.008**	11.745	1.000	**0.027**	**0.024**	0.411	0.647	1.000
O1	**<0.001**	18.193	1.000	**0.001**	**0.032**	0.118	0.672	1.000
O2	**0.001**	17.719	0.390	**<0.001**	**0.049**	1.000	0.693	1.000
T6	**0.001**	16.368	0.470	**0.020**	**0.001**	1.000	0.852	1.000

EEG δ, θ, and α absolute power across consciousness states. P-value and χ refer to Kruskal-Wallis tests with grouping variable set to the consciousness state and independent variable to the PSD of the specific electrode. Pair-wise comparisons results derive from Dunn post-hoc tests, Bonferroni corrected. Variables in bold are considered significant.
