# Supplementary figures and images for: Neural coding of autonomic functions in different states of consciousness
Source: J Neuroeng Rehabil. 2023 Jul 26;20:96. doi: 10.1186/s12984-023-01216-6 (PMC10369699; doi:10.1186/s12984-023-01216-6)

Supplementary Materials C

Reproduction of Figure 2 and 3 with referencing to Fpz.


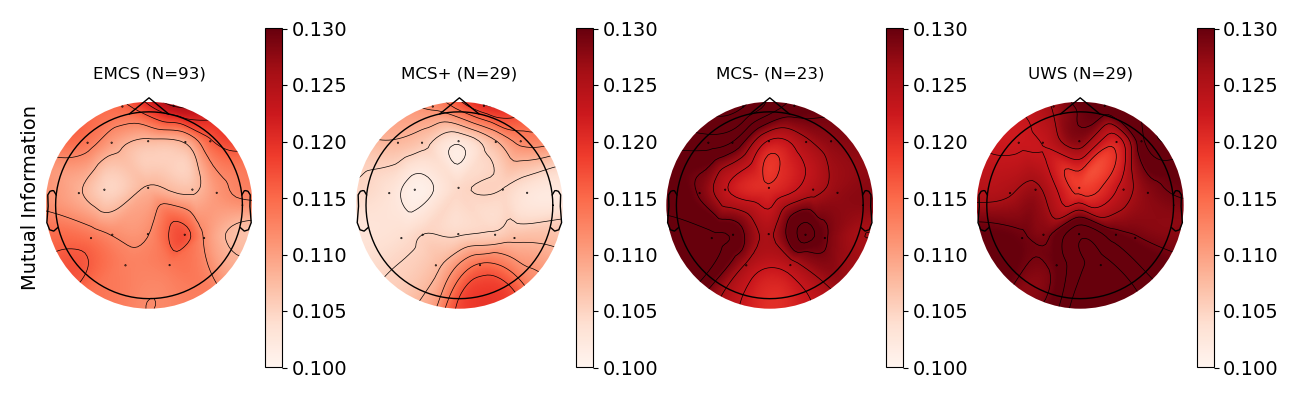


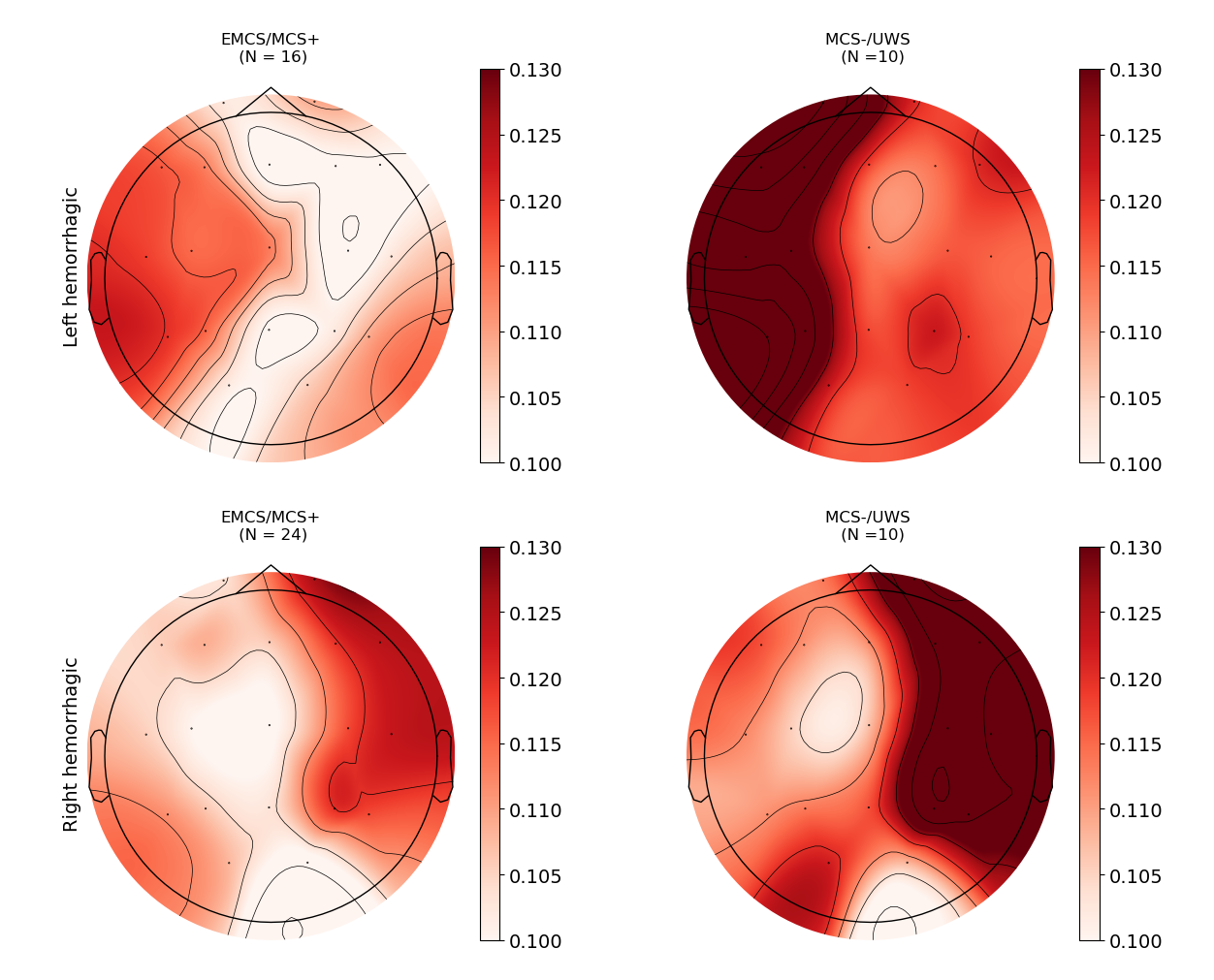

Supplement: Supplementary file 4 — Additional file 4. Reproduction of Figures 2 and 3 with re-referencing to Fpz. [file 12984_2023_1216_MOESM4_ESM.docx]
